# Monosialoganglioside GM1 reduces toxicity of Ptx and increase anti-metastasic effect in a murine mammary cancer model

**DOI:** 10.1038/s41598-020-67256-3

**Published:** 2020-06-23

**Authors:** Victoria Leonhard, Roxana V. Alasino, María E. Pasqualini, David C. Cremonezzi, Néstor H. García, Dante M. Beltramo

**Affiliations:** 1 Centro de Excelencia en Productos y Procesos de Córdoba (CEPROCOR), Córdoba, Argentina; 2 Consejo Nacional de Investigaciones Científicas y Técnicas (CONICET), Córdoba, Argentina; 3 Instituto de Investigaciones en Ciencias de la Salud- FCM (INICSA-CONICET), Córdoba, Argentina; 4 Cátedra de Patología - Hospital Nacional de Clínicas - Universidad Nacional de Córdoba, Córdoba, Argentina; 5 Facultad de Ciencias Químicas - Universidad Católica de Córdoba, Córdoba, Argentina

**Keywords:** Cancer, Medical research, Oncology

## Abstract

Having demonstrated the ability of monosialoganglioside GM1 micelles as oncology drug transporter, this work focuses on evaluating its application in an *in vivo* system, studying the toxicity and antitumoral effect of GM1-Ptx micellar formulation. The maximum tolerated dose (MTD) obtained after intravenous administration of GM1-Ptx in mice was 55 mg/kg and the 50% lethal dose (LD50) was 70 mg/kg. This value is higher than those described for the commercial formulations TAXOL and ABRAXANE, with LD50 of 30 and 45 mg/kg respectively. The antitumor activity, mortality and incidence of metastasis were studied on a murine model of mammary gland cancer. The GM1-Ptx formulation was administered i.v. at different doses for 9 weeks using empty GM1 micelles and saline as treatment controls. Once the treatments were completed, biochemical markers were quantified and histological tissue tests were performed. The most promising results were obtained with the treatment at a dose of 15 mg/kg/twice a week, condition in which a longer survival and significant reduction in the incidence of animals with metastasis, since only one 25% of the mice showed presence of pulmonary micro metastases.

## Introduction

Despite the great advances achieved in cancer therapy, the achievements in relation to advanced or metastatic malignancies are very few. Patients with metastatic disease have a poor prognosis, even after successful management of the primary tumor^1^. The factors involved in the formation, progression and metastasis of tumors are multiple and not yet fully understood^2^.

Paclitaxel (Ptx) is one of the most commonly used antitumor drugs today for several cancers^3^. Due to its low solubility in water, this drug was formulated in a mixture of 50% CREMOPHOR EL (polyethoxylated castor oil) and 50% dehydrated ethanol, a commercial formulation known as TAXOL^4^. The large amount of solvent used for this formulation produces numerous side effects^5–7^. In order to avoid toxicities associated with co-solvents, many laboratories have developed new strategies to increase the aqueous solubility of Ptx. To date, only ABRAXANE has been shown to decrease the toxic effects of TAXOL^8–10^. However, the preparation of this formulation is quite complex and also has low stability once reconstituted in saline (a maximum of 8 h refrigerated at 2 °C to 8 °C) therefore, the reconstituted powder must be rapidly injected into the patient^8^. For these reasons, alternative formulations for paclitaxel are still actively pursued.

Previously, we demonstrated that monosialoganglioside (GM1) micelles spontaneously load high amounts of Ptx resulting in a very stable nanostructure^11^. *In vitro*, Ptx loaded into GM1 micelles showed the same pharmacological effect than the free drug^11^. On the other hand, the GM1-Ptx complex is also able to interact with albumin in a non-covalent way to render a tertiary GM1-Ptx-Albumin complex^12^.

In addition to the intrinsic proliferative capacity of the tumor cells, the interaction with their microenvironment is a critical factor for their growth and for the inhibition of the antitumor immune response, which leads to a later spread and implantation of the tumor in a new site^13,14^. About the inhibition of the immune response, while several of the mechanisms employed by cancer cells are known, myeloid-derived suppressor cells (MDSCs) are one of the major drivers of tumor-mediated immune evasion. These are immature myeloid cells that are recruited to the TME where they become very powerful immunosuppressive cells.

Many approaches have been investigated in order to inhibit the action of the MDSCs;^15–20^ yet, results were not successful, it being a challenge to find a suitable molecule for cancer treatment^21^. It has recently been discovered that gangliosides secreted by tumor play an important role in the recruitment and interaction with MDSC^22^, producing a blockade of host immune function by multiple mechanisms^23,24^. Also many studies have shown that those tumors whose cells contain and shed a large amount of gangliosides to the TME are more tumorigenic^25,26^ and have a higher metastatic potential^27,28^ than tumors with lower contents of these lipids.

The fact that gangliosides are able to carry antitumor drug such as Ptx, as well as interact with MDSC, lead us to design a new antitumor strategy based on that GM1-Ptx complex. Our hypothesis is that GM1-Ptx complex would be able to reach the site of the tumor and kill tumor cells, while on the other hand would interact with MDSC and also eliminate them, leading to reactivation of the immune system and decreasing tumor cells growth.

In this work, we studied the *in-vivo* toxicity of GM1-Ptx micelles and the effect on tumor growth and metastasis formation in a murine mammary gland cancer model.

## Results

### Single-dose toxicity study

**Figure 1:**
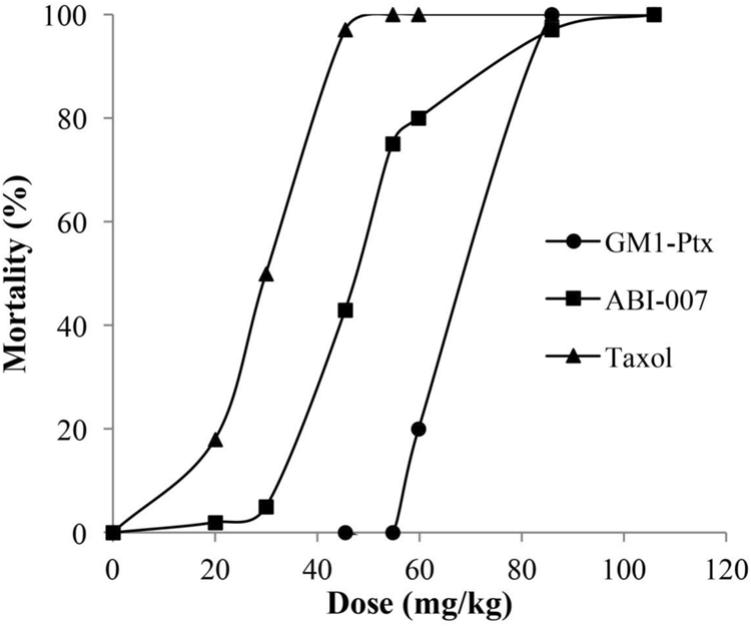
LD50 for GM1-Ptx formulation compared against doses reported for Cremophor-based paclitaxel (TAXOL) and Albumin-based paclitaxel (ABI-007).

The first objective of this study was to evaluate the *in vivo* toxicity of the GM1-Ptx formulation compared to the reference ones. Figure 1 shows the LD50 results from the treatment of mice with GM1-Ptx, with respect to the reported values of the two existing commercial formulations, TAXOL and ABRAXANE (ABI-007)^29^. The administration of a single dose of GM1-Ptx at a concentration of 55 mg/kg produced no mortality after 14 days of observation, while the lethal dose of 50% (LD50) was 70 mg/kg. Since LD50 for TAXOL and ABRAXANE are 30 and 47 mg/kg, respectively, these results suggest that the GM1-Ptx formulation is significantly less toxic than the reference formulations.

### Effect of GM1-Ptx on clinical and biochemical blood parameters during tumor development

Here we evaluate clinical and biochemical parameters that are generally considered relevant, since their changes during tumor development reflect the prognosis. Animals were separated into five groups, where group 1 only receive saline, group 2 receive empty GM1 micelles and groups 3, 4 and 5 receive GM1-Ptx with different doses of drug, during nine weeks of treatment. The first clinical parameters evaluated at the end of treatments were the weight of animals and the size of tumor developed under each conditions. Table 1 shows that Group 1 (control) mice had a weight loss of 1.1 ± 1.1% at the end of treatment, while mice treated with empty GM1 micelles (Group 2) had an increase in weight of 13,9 ± 1,4%. With respect to the treatments with Ptx at different doses and administration regimens, the most favorable result was observed in Group 4 (15.8 ± 0.9%), in which 30 mg/kg of Ptx were injected in two weekly doses of 15 mg/kg each. Then in Group 5, where the same total amount of Ptx (30 mg/kg/week) was injected, but in a single weekly dose, showed an increase of only half the weight reached by the animals in group 4. Finally, in Group 3, which received half of the total mass of Ptx (15 mg/kg/week), the weight gain was significantly lower in relation to Group 4 (15.8 vs 11.1). Table 2 also shows the changes in the weight of the primary tumor and the Bw/TW index. Results were similar to those found for the weight of the animal, with Group 4 showing the smallest primary tumors (p < 0.026) and a higher BW/TW index (p < 0.001)^30^. These results show that the best conditions for the treatment of tumors with GM1-Ptx were 15 mg/kg twice a week.

**Table 1:** Effect of the treatment with GM1-Ptx micelles on tumor-bearing mice.

Description	Group 1	Group 2	Group 3	Group 4	Group 5
	Control Tumor	Empty GM1 2/week	GM1-Ptx 15 mg/kg 1/week	GM1-Ptx 15 mg/kg 2/week	GM1-Ptx 30 mg/kg 1/week
Ptx doses (mg/kg/week)	0	0	15	30	30
Initial body weight (g)	26,2 ± 0,6	22,3 ± 0,8	22,6 ± 0,9	23,4 ± 0,5	27,7 ± 0,9
Final body weight (g)	25,9 ± 0,5	25,4 ± 0,6	25,1 ± ,0,4	27,1 ± 0,4	29,7 ± 0,8
Difference in weight (%)	−1,1 ± 1,1	13,9 ± 1,4	11,1 ± 1,3	15,8 ± 0,9	7,2 ± 1,7
Primary tumor weight (g)	4,1 ± 0,5	3,3 ± 0,4	3,0 ± 0,3	2,6 ± 0,2*	3,8 ± 0,4
BW/TW index (g)	6,3 ± 1,1	7,7 ± 1,0	8,4 ± 0,7	10,4 ± 0,6*	7,8 ± 1,2

**Table 2:** Biochemical changes in serum parameters of tumor-bearing mice treated with GM1-Ptx.

	Reference Values	Group 1	Group 2	Group 3	Group 4	Group 5
		Control Tumor	Empty GM1 2xweek	GM1-Ptx 15 mg/kg 1/week	GM1-Ptx 15 mg/kg 2/week	GM1-Ptx 30 mg kg 1/week
Albumin (g/dL)	3.5–4.6*	2.1 ± 0.1	2.2 ± 0.1	2.1 ± 0.2	2.3 ± 0.1	2.4 ± 0.2
Creatinine (mg/dL)	0.15–0.2*	0.20 ± 0.03	0.20 ± 0.20	0.10 ± 0.01	0.20 ± 0.02	0.20 ± 0.04
AST (UI/mL)	135–352*	287 ± 33	221 ± 48	186 ± 19	216 ± 33	345 ± 12
ALT (UI/mL)	60–126*	17 ± 3	73 ± 8	11.0 ± 1.5	36.0 ± 5.1	32.0 ± 3.2

^*^Reference values^55^.

It is known that several biochemical parameters like serum proteins levels (albumin), or enzymes such AST and ALT and metabolites like creatinine usually change during course of disease. Here, in Table 2, we show the effect on biochemical parameters of tumor-bearing mice during the treatment with GM1-Ptx micelles. Considering the normal values, it can be observed that the animals of the five groups showed a marked decrease in serum albumin, but not in the rest of the parameters measured, which were not affected by the tumor or by the treatment with GM1-Ptx.

**Figure 2:**
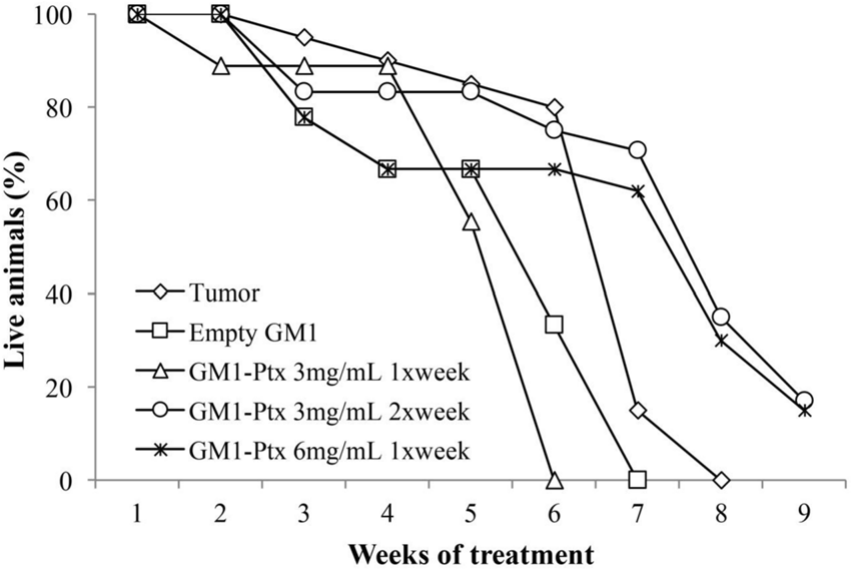
Effect of the treatment with GM1-Ptx micelles on survival of tumor-bearing mice.

On the other hand, under similar experimental conditions, we evaluate the mice’s life span. Figure 2 shows that the survival of the Groups 2 and 3 mice in the seventh week of treatment is 0 (zero), while the survival of the animals in Groups 4 and 5 is approximately 8 times higher than in the control group of mice treated only with saline. In addition, 17% of Group 4 animals and 15% of Group 5 animals remained alive at the end of the experiment at week 9. The results show that high doses of GM1-Ptx (30 mg/kg) increase the survival of mice.

It is well known that the survival of tumor-bearing animal depends on tumor metastasis, thus, we evaluated the incidence of macrometastasis in lungs, liver and kidneys and also the presence of micrometastasis in lungs. The results of Table 3 show that in Group 1, where the mice were treated only with saline, the incidence of pulmonary macrometastases appears in 40% of the animals, while micrometastases are observed in 83% of the mice. In liver, macrometastasis was detected in only 5% of mice, whereas in kidney no macrometastsis was observed in any mice. Animals of Group 2, treated with empty GM1 micelles, show macro and micrometastases in 56% and 100% of mice respectively, representing an increase of 21.7% and 19%, respectively, of animals with metastasis. Liver and kidney analysis shows that empty GM1 micelles induced hepatic macrometastasis in 33% of mice, but, as in Group 1, none in kidney. These results show that empty GM1 micelles have the ability to induce an increase in tumor metastasis. When mice were treated with GM1-Ptx formulations in different administration protocols, only 22% of Group 3 animals (15 mg/kg/week) developed pulmonary macrometastases. On the other hand, histological studies showed that 100% of the mice of Group 3 (15 mg/kg/week) and Group 5 (30 mg/kg/week) developed micrometastases in this organ, unlike Group 4 where the percentage of affected mice reached only 25%.

These results show that although it was possible to eliminate 100% of hepatic and renal macrometastases in the three treatment protocols tested (Groups 3, 4 and 5), only Group 4 showed a significant reduction in the incidence of macro and micrometastases.

**Figure 3:**
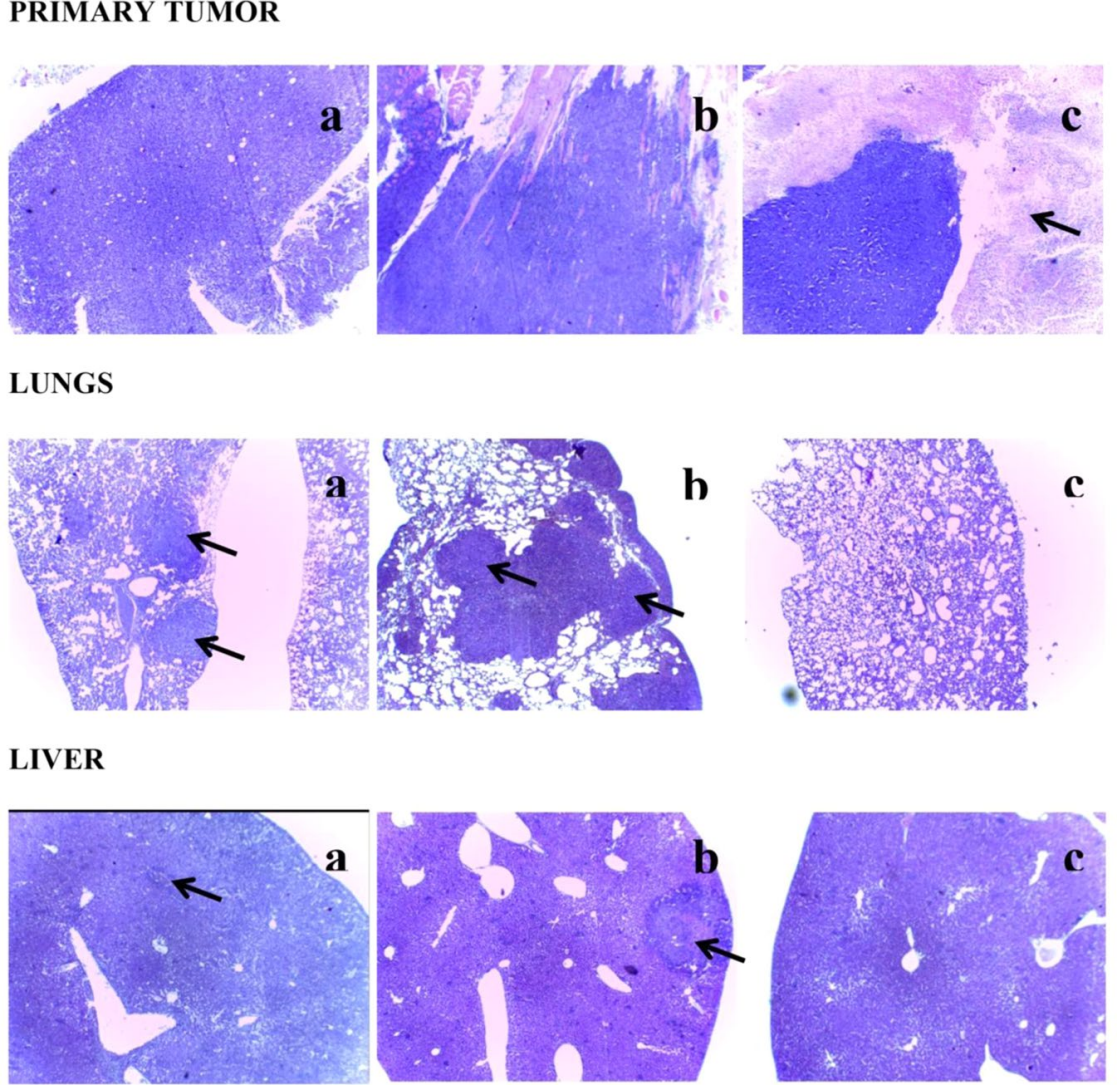
Histological sections at the primary tumor (upper panel), lungs (middle panel) and liver (lower panel) of tumor-bearing mice of Group 1 (**a**), Group 2 (**b**) and Group 4 (**c**) at low magnification (40×).

Figure 3 shows the histological sections performed of the primary tumor and also of the lung and kidney before and after treatments with GM1-Ptx. The images correspond to organs of mice of Group 1 (a), Group 2 (b) and Group 4 (c). The upper panel shows the primary tumors in muscle; the middle panel, the lung sections and the lower liver panel. Groups 1 and 2 show the primary tumor already implanted surrounded by compressed muscle fibers; while in Group 4 (c) animals, extensive areas of necrosis are observed around the tumor cells as an effect of the GM1-Ptx treatment. In the lungs of the mice of groups 1 and 2, multiple nodules of metastatic tumors are observed, while they are found very infrequently in animals of group 4, with very similar appearance to the norm, with respiratory tracts that preserve their epithelial lining and the usual thickness of the walls. In the liver, a few metastatic tumor nodules are observed in Groups 1 and 2 mice, unlike Group 4 animals that do not show metastasis in this organ.

**Table 3:** Effect of the treatment with GM1-Ptx micelles in the incidence of metastases in tumor-bearing mice.

	Group 1	Group 2	Group 3	Group 4	Group 5
	Control Tumor	Empty GM1 2xweek	GM1-Ptx 15 mg/kg 1/week	GM1-Ptx 15 mg/kg 2/week	GM1-Ptx 30 mg/kg 1/week
Ptx doses (mg/kg/week)	0	0	15	30	30
% of animals with metastases					
Pulmonary macrometastases	40	56	22,2	0	0
Pumonary. micrometastases	84	100	100	25	100
Hepatic macrometastases	5	33	0	0	0
Renal macrometastases	0	0	0	0	0

In general, gangliosides are considered immunologically tolerated autoantigens to a greater or lesser degree that are classified as T-cell independent antigens, i.e, they are not recognized by T lymphocytes and, therefore, do not provide cytokines to promote maturation of the system immune^31^. Notwithstanding, there are numerous reports about the generation of anti-ganglioside autoantibodies and also about their implication in different pathologies^32,33^. On the other hand, because many tumor cells release immunosuppressive gangliosides to TME, some authors have explored their use as immunological targets^34^. In mice, it has been described that GD3 and GM1 are the most immunogenic gangliosides within the family^35^. Considering that the treatment used in this work consisted of the injection of GM1 ganglioside nanocarriers of porcine origin in tumor-bearing mice, a potential immunological response of the animals against the exogenous GM1 applied was evaluated. In this sense, the results in Table 4 showed that there was no generation of anti-GM1 antibodies, IgM and IgG, at least until week 8 after treatment; thus, it can be inferred that the immune component does not present a risk factor in the treatment.

**Table 4:** Generation of anti-GM1 antibodies in in tumor-bearing mice treated with GM1-Ptx micelles. Values are the average of at least three determinations, SEM was less than 0.6% in all cases.

Abs 450 nm	0 weeks	3 weeks	4 weeks	8 weeks
IgM anti-GM1	0.24 ± 0.02	0.17 ± 0.03	0.24 ± 0.04	0.24 ± 0.03
IgG anti-GM1	0.01 ± 0.01	0.02 ± 0.01	0.01 ± 0.02	0.01 ± 0.02

**Figure 4:**
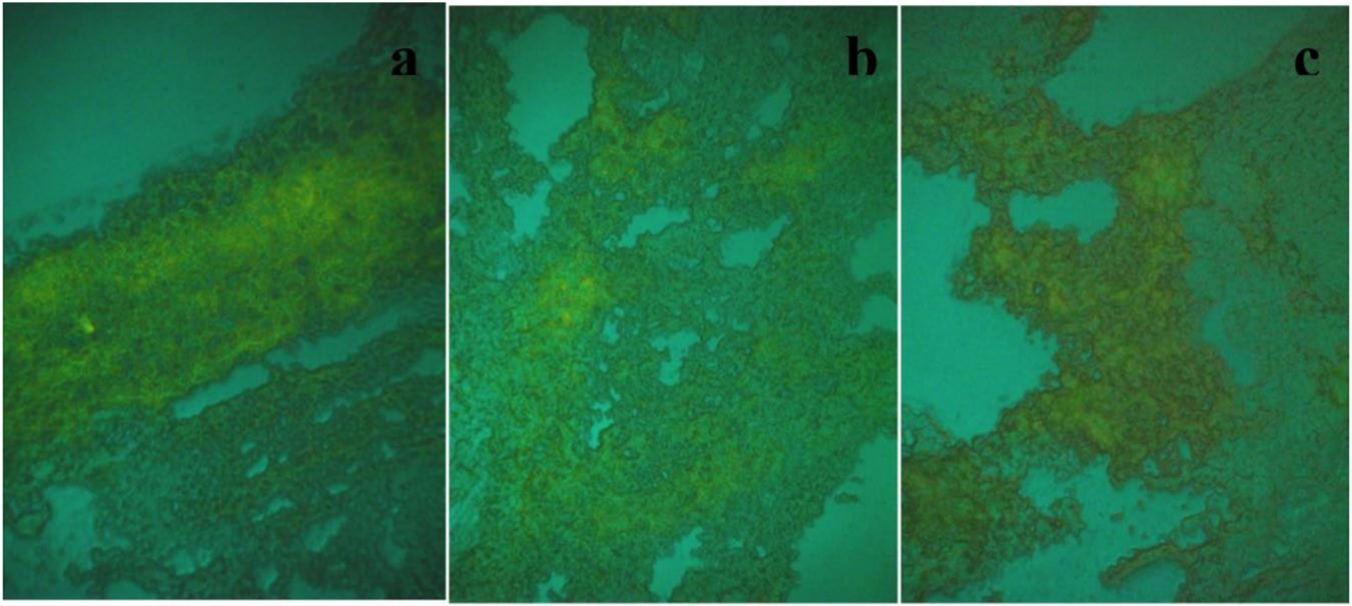
Histological sections of the primary tumor after 12 (**a**), 24 (**b**) and 48 (**c**) h post-injection of the fluorescent formulation.

Moreover, to demonstrate whether GM1 micelles reach the tumor site, these were labeled with a fluorescent dye and administered to mice by the lateral tail vein, during a short period of isoflurane-induced inhalation anesthesia. Figure 4 shows a homogenous distribution of labeled GM1 along the tissue. A quantitative analysis of the observed fluorescence was not performed, however, it seems that there is a slight decrease in the intensity of the fluorescence from 12 to 24 hours and towards 48 hours.

**Figure 5:**
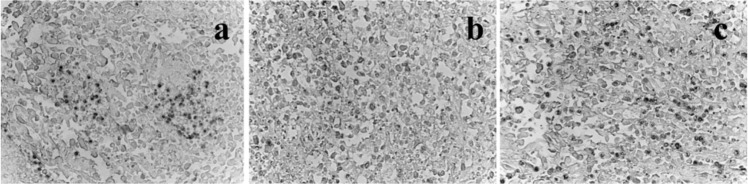
Histological sections of the primary tumor of mice of Group 1 (**a**), Group 2 (**b**) and Group 4 (**c**) at 400× magnification.

In addition, cell death assays were performed with TUNEL staining in histological sections of the primary tumor of mice after different treatments. Figure 5 showed staining of tumors of Group 1 mice without treatment (a), of Group 2 treated with empty GM1 micelles (b) and of Group 4 treated with GM1-Ptx micelles 15 mg/kg twice a week (c). It observed that mice of Group 2 have 40% less number of apoptotic cells per field than the control group without treatment; while the animals of Group 4 have 20% more apoptotic cells than untreated mice.

Considering the fact that the treatment of tumor-bearing mice with empty GM1 micelles resulted in a worse prognosis for the animals, with a decrease in survival and an increase in the amount of metastasis, and, on the other hand, knowing the studies carried out by Ladisch et.al., who demonstrate the relationship between gangliosides in TME and the recruitment of MDSC cells, with strong associated immunosuppressive activity, we evaluate what happens with these cells in the different treatments evaluated. To detect the presence of MDSCs on the primary tumor, a kit of monoclonal antibodies specific for CD11b, Gr-1 and Ly6G markers was used. Originally MDSCs were described in mice by the co-expression of two markers, CD11b and Gr-1. However, later studies demonstrated that this population was not homogeneous due to the presence of different groups of myeloid cells with different suppressive activity. At present, at least two subtypes of MDSCs in tumor-bearing mice are known, monocytic (M) and granulocyte (G) types. These are identified by the differential expression of Ly6C and Ly6G, the two Gr-1 isoforms, being G-MDSC positive for Ly6G and M-MDSC for Ly6C^36^.

**Figure 6:**
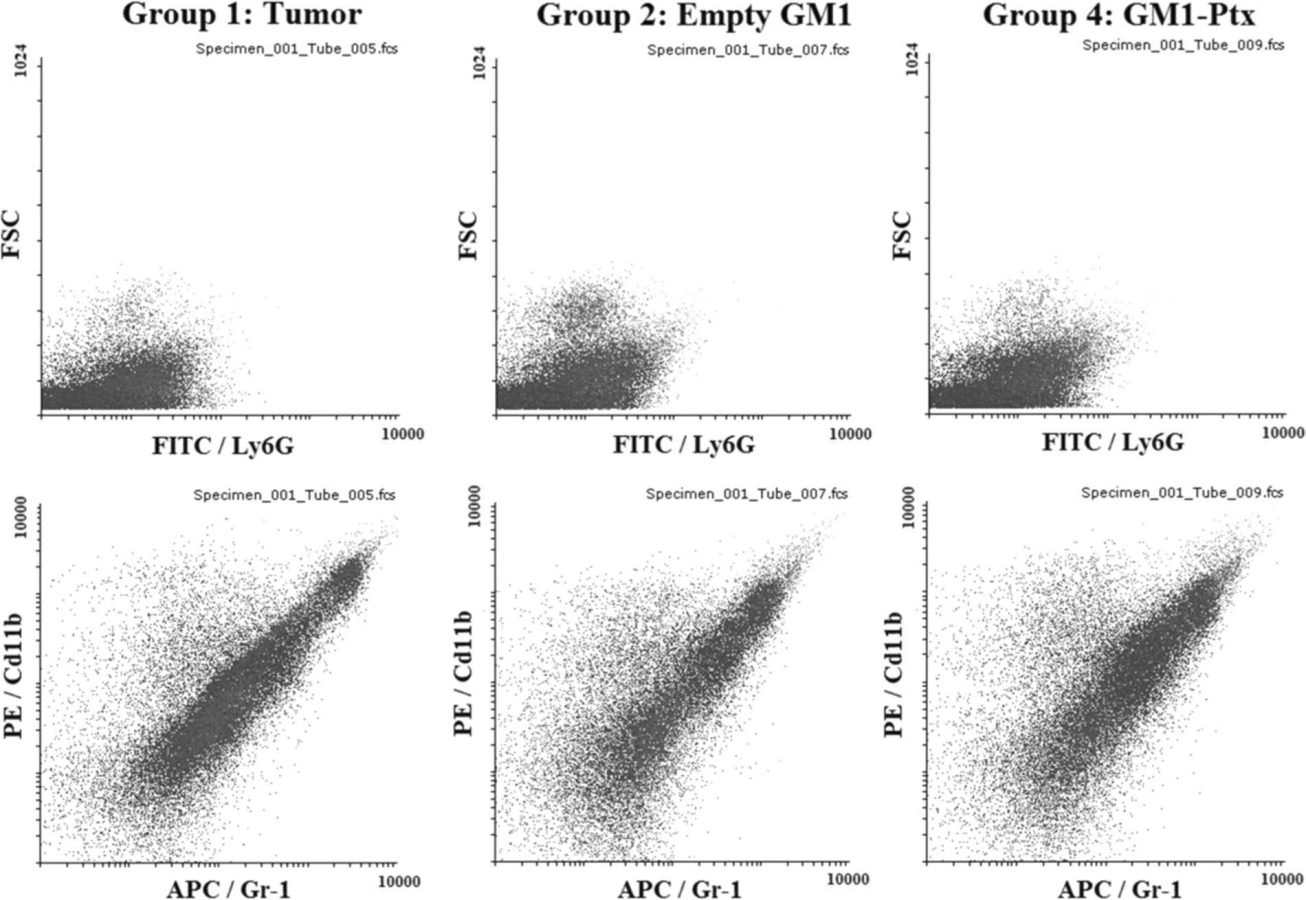
Dot plots showing distribution of MDSC on primary tumor of mice under the different treatments.

Figure 6 shows MDSC’s plot distribution on a primary tumor of mice from Groups 1, 2 and 4. It is observed that in treatments with GM1 micelles, either empty or loaded with Ptx, produce an increase in the population of APC/Gr-1 and PE/CD11b cells that can be seen at the level of the primary tumor, more evident in GM1-Ptx micelles.

Our studies showed negative results for FITC/Ly6G marker which is why we estimated that the population of immature cells present in our system corresponds to M-MDSC.

## Discussion

We have previously demonstrated the ability of GM1 micelles to solubilize large amounts of Ptx by a simple procedure. These micellar structures, with a size in the order of 10 nm, proved to be very stable in aqueous solution, for up to 4 months maintained at 4 °C to 8 °C, and also in solid states after a lyophilization process. In addition, we have shown that this micellar system is suitable for transporting various kinds of hydrophobic and hydrophilic drugs and is even capable of loading more than one drug in the same micelle due to the existence of micro-domains of different polarities in this structure^37,38^. Also, other authors have demonstrated that GM1 micelles can cross the blood-brain barrier and therefore would have a great potential for the administration of drugs to treat intracranial diseases such as brain tumors and neurodegenerative diseases^39^.

A single-dose toxicity study, with a 14-day follow-up, showed that MTD and LD50 of GM1-Ptx formulation were 55 mg/kg and 70 mg/kg, respectively. These values were significantly higher than those of the commercial formulations TAXOL and ABRAXANE, demonstrating that the micellar formulation has lower toxicity, which would allow higher doses than those used in the current drug administration protocols.

The antitumor efficiency of GM1-Ptx formulation in BALB/c mice with a highly metastatic tumor induced was then evaluated. Different administration regimes of this formulation as well as the micellar carrier alone were studied and tested against saline to discriminate between specific GM1 toxicity and Ptx-induced tissue injury. It was chosen to work with doses of drugs lower than the maximum tolerated dose of the formulation, corresponding to 15 and 30 mg/kg/week to be able to compare against bibliographic values obtained for the commercial formulations TAXOL and ABRAXANE.

The treatment with 15 mg/kg/week of GM1-Ptx formulation was not enough to improve the survival of mice with tumors in the seventh week of treatment, while treatments with doses of 30 mg/kg/week managed to increase survival of animals, reaching around 60% in the same period. Group 4 mice, with a GM1-Ptx treatment of 30 mg/kg/week but separated into two weekly doses of 15 mg/kg/week, showed a significant reduction in primary tumor size (> 35%) and weight gain body throughout the treatment. It should be noted that the incidence of pulmonary micrometastases in this group affected only 25% of the animals, compared to 100% observed in the other treatment schemes. Group 2 mice, treated with empty GM1 micelles, have been shown to have a worse prognosis than those treated with saline alone. The treatment with the vehicle has greatly reduced the survival of diseased mice and has increased the incidence of pulmonary and liver macrometastases. These results are consistent with that described by Ladisch et. al., who demonstrated a close relationship between gangliosides and the immune escape and tumor outgrowth, through the increase of infiltrating myeloid-derived suppressor cells (MDSC)^14,23–26^. They hypothesize that some tumors produce and release gangliosides to TME to evade the immune system and that the mechanism involved would be through the recruitment of MDSC cells. In this regard, flow cytometry studies show an increase in the population of MDSC cells (Cd11b and Gr-1) present in TME with GM1 treatment, with respect to mice treated with saline.

In the treatment with GM1-Ptx twice a week (Group 4), an increase in the population of cells CD11b and Gr-1 in the TME is also observed, although in this case, it was associated with a higher survival rate, a decrease in the primary tumor size and a significant reduction in the amount of metastasis. This could imply a change in the suppressive activity of the MDSC given by a different population of these immune cells, as described Sierra et.al. and Bronte et.al.^40,41^. However, we cannot confirm this on the basis of the tests carried out.

The increase in MDSC could also respond to a transient effect on the TME induced by the presence of GM1. However, these would not be functionally active due to the effect of the Ptx contained within the micelles, which would ultimately allow preserve the mouse immune function.

The pathological anatomy of the organs of animals of the different groups evaluated shows great difference according to the treatment received. On the other hand, cell death assays, carried out by the TUNNEL technique, corroborate not only the activity of the GM1-Ptx formulation, but also the effect of GM1 to enhance tumor formation by the inhibition of mechanism of apoptosis, previously described by other authors^42–44^.

An important improvement in the GM1 micelle system could be ascribed to the sialic groups present in the ganglioside molecules. The participation of sialic acid (SA) and its derivatives in immune regulation is currently recognized as one of the most important axes in the health-disease balance. We can find a wide variety of works on the use of SA as surface modifiers of nanocarriers with the aim of improving their characteristics of transport and targeted drug delivery^45^. In this sense, the use of GM1 was one of the modifiers first proposed, mainly as a stabilizer of nanotransporters. The first works date from the late 1980s, from which numerous reports have emerged^46,47^.

Whether from improvement as a transport system or from the advantages associated with the increase of antitumor immunity by blocking the binding of the SA groups of the tumor cells to the Siglecs (sialic-acid-binding immunoglobulin-like lectins), GM1 micelles constitute one of the current alternatives of greater potential for the treatment of cancer^48,49^.

The results showed that the treatment of mice inoculated with highly metastatic tumor cells with GM1-Ptx micelles (Groups 4, with doses of 30 mg/kg/week) produced a significant increase in survival and a decrease in metastasis in target organs, as well as a considerable improvement in clinical markers, compared to mice untreated, treated with low doses or treated with empty GM1 micelles.

An important finding in this study was that the treatment of tumor-bearing mice with GM1-Ptx micelles (Group 4) not only managed to decrease the number of animals with metastases to 25%, but also reduce the size of the primary tumor up to 35%.

In addition, DL50 and MTD were much higher for this formulation than for commercial TAXOL and ABRAXANE. Therefore, new administration regimes could be established using more doses and producing less toxic effects.

## Methods

Monosialogangliosides GM1 from pig brain was a gift from TRB-Pharma S.A. (Buenos Aires, Argentina); Paclitaxel (Ptx) was obtained from Sigma Chem Co. The GM1-fluorescent complex was prepared by direct encapsulation of the fluorescent dye FITC into GM1 micelles.

Murine mammary adenocarcinoma cell line (LMM3) with high metastatic capability was kindly provided by Dr. Bal de Kier Joffe E from Roffo Hospital (Buenos Aires, Argentina). This cell line was syngeneic to BALB/c mice derived from spontaneous lung metastasis from the primary MM3 mammary tumor^50,51^.

Eighty-eight weaning male BALB/c mice (7 weeks old, 20–25 g) were randomized and housed in polycarbonate cages in groups of four, in a 12-h light and 12-h dark cycle at a constant temperature of 23 °C and fed with a pelletized diet and tap water ad libitum prior to and during experimental procedures. Animal studies were conducted in accordance with the guidelines set by the National Institutes of Health (NIH) Guide for the Care and Use of Laboratory Animals (USA) and approved by the Institutional Committee for the Care and Use of Laboratory Animals at the School of Medical Sciences (Universidad Nacional de Córdoba, Argentina).

### Standard procedure for preparing GM1-Ptx micelles

Stock solutions of GM1 with a concentration of 250 mg/mL were prepared in bidistilled water as described in Leonhard *et al*.^11^. Stock solutions of Ptx were prepared in dimethylsulfoxide (DMSO) at a concentration of 50 mg/mL. This solution was added to GM1 micellar solutions until achieving a Ptx concentration of 3 or 6 mg/mL. The mixtures were stirred for 2 min and then incubated at 4 °C for 24 h before dialysis for 24 h at the same temperature against 100 volumes of bidistilled water to remove all DMSO.

### Single-dose toxicity study

Twenty healthy animals were divided randomly into five groups containing 4 animals each. The animals were administered different doses of GM1-Ptx formulation (45, 55, 60, 85 and 105 mg/kg). The animals were monitored for 14 days for mortality, clinical and behavioral symptoms, and any adverse reaction.

### Efficacy study

#### Tumor cell culture

Cancer LMM3 cells were grown in MEM (Gibco, BRL) supplemented with 3 mM L-glutamine, 80 μM/mL gentamycin and 10% fetal bovine serum (NATOCOR, Córdoba, Argentina) at 37 °C with 5% CO_2_.

#### *In-vivo* tumor induction

BALB/c mice were injected intramuscularly in the right thigh with 0.1 mL of LMM3 cell suspension (1 × 10^6^ cells in MEM without serum and antibiotics) to generate the primary tumor. After tumor induction, mice were housed until palpable tumor was detected (7 days)^52^. Animals with tumor developed were included for subsequent experiments. For survival determination, induced death or spontaneous death was considered as an end point, based on the Georgia Regents University protocol for animal research in solid tumor production and cancer^53^.

#### GM1-Ptx treatment

Ten days post inoculation, when tumor was palpable^52^ and reached approximately a volume of 100 mm^3^, mice (n = 50) were randomly divided into 5 groups and different formulations were injected by the lateral tail vein, during a short period of isoflurane-induced inhalation anesthesia. All animals were followed for 10 weeks or until the end point was reached.

The experimental groups (n = 10 each) were:

Group 1: Control tumor progression. Injection of 100 µL saline solution twice a week.

Group 2: Injection of 100 µL GM1 solution dissolved in saline at a concentration of 108 mg/mL twice a week.

Group 3: Injection of 100 µL of GM1-Ptx solution dissolved in saline at a concentration of GM1 = 108 and Ptx = 3 mg/mL once a week.

Group 4: Injection of 100 µL of GM1-Ptx solution dissolved in saline at a concentration of GM1 = 108 and Ptx = 3 mg/mL twice a week.

Group 5: Injection of 100 µL of GM1-Ptx solution dissolved in saline at a concentration of GM1 = 250 and Ptx = 6 mg/mL once a week.

#### Biological and biochemical tumor host mice determinations

Tumor growth was measured with calipers 10 days after injection of tumor cells and at the end of the trial. All mice involved in the study were weekly weighed. After 10 weeks, the surviving animals were sacrificed by lethal anesthesia. Daily animals were followed by completion of end point.

Blood samples were collected by intracardiac puncture on ethylene-diamine-tetraacetic acid (EDTA) tubes from mice at the end of the experimental period. After centrifugation of whole blood, serums were obtained to evaluate different biochemical indicators of hepatic and renal pharmacological toxicity (enzymes, proteins and hepatic metabolites). These analyses were done at Laboratorio de Alta Complejidad Córdoba (LACE) (Córdoba, Argentina).

#### Histopathological analysis

During necropsy tumor weight, size and presence of macro metastasis in kidney, lung and liver were evaluated. The weight of the primary tumor was later corrected by body weight of each animal and analyzed as index of tumor growth.

In addition, the presence of micro metastasis in lungs was analyzed. For that, at least 8 tissue lung sections of each animal were evaluated. All tissues were fixed in 4% phosphate-buffered formalin then embedded in paraffin, and 5-μm sections were stained with haematoxylin-eosin.

The pathologist performed all the microscopic evaluations without knowledge of the treatment received by each of the animals.

### Generation of anti-GM1 antibodies

Detection of IgM or IgG anti GM1 in mouse serums was conducted according to Mizutamari *et al*.^54^. Briefly, samples of GM1 (300 ng on 50 µL of Methanol) were dried on a 96 well Petri dish for 24 h at 25 °C. Then, 200 µL per well of a solution of 1% bovine serum albumin (BSA) in PBS were adding to block the free binding site before incubating for 1 h at 4 °C. Subsequently 50 µL per well of mouse serum samples diluted from 1/10 to 1/100 with PBS were incubated with GM1 for 24 h at the same temperature. The vials were then washed with PBS and incubated with 50 µL per well of the secondary rabbit antibody peroxidase-labeled anti mouse IgG or IgM for 2 h at 4 °C.

After further washing with PBS, samples were added 150 µL per well of a solution containing the enzyme substrate solution consisting of 3.7 mM to orthophenylenediamine (OPD) in phosphate/citrate buffer pH 5 (80/35 mM) and H_2_O_2_ 0.02%. Incubation was performed for 30 min at 37 °C. After that, the reaction was stopped with 75 µL per well of a H_2_SO_4_ 0.5 N solution and product absorbance was read at 450 nm.

### Injection of fluorescent-GM1 and histopathological analysis

To investigate how fast GM1 micelles arrive to the primary tumor site, 10 days post inoculation, when tumor reached a volume of approximately 100 mm^3^, 3 groups of 3 mice were slowly i.v. injected with a GM1-fluorescent by the lateral tail vein, during a short period of isoflurane-induced inhalation anesthesia. After 12, 24 or 48 h post injection, animals were sacrificed. Once isolated the primary tumor, cut sections 5–15 µm thick were obtained using a cryostat at −20 °C. Within 1 min of cutting the tissue sections were moved to a poly-L-lysine-coated slides to improve adherence and then immediately immersed into a fixative (cryo plast) for evaluation at room temperature using a fluorescence microscope.

### *In-Vivo* Apoptosis Assays

Apoptosis was determined by the terminal deoxyribonucleotidyltransferase-mediated dUTP nick-end labeling (TUNEL) method using the DeadEnd Colorimetric TUNEL System (Promega, Madison, WI). Tumor tissue sections from 3 animals of groups 1, 2 and 4 were selected randomly and they were observed in 10 random fields under a light microscope at 400× magnification.

### Flow-cytometer analysis

Detection of MDSC cells was performed using specific mouse monoclonal antibodies against CD11b, Gr1 and Ly6G markers Mouse MDSC Flow Cocktail 1 with Isotype Ctrl from Bio Legend. For this purpose we used 3 mice from each of the following groups:

Group 1: Injection of 100 µL of saline solution twice a week for a total of 5 doses.

Group 2: Injection of 100 µL GM1 solution dissolved in saline at a concentration of 108 mg/mL twice a week for a total of 5 doses.

Group 4: Injection of 100 µL of GM1-Ptx solution dissolved in saline at a concentration of GM1 = 108 and Ptx = 3 mg/mL twice a week for a total of 5 doses.

Seven days after final dose, animals were sacrificed and primary tumors were washed with PBS solution and then disrupted with a scalpel. 10 mL of 0.01% Pronase and 0.24% DNase (500 µL) enzymes were added to each tumor. The solution was stirred in an oven at 37 °C for 45 min and then filtered. Cell count on the supernatant was then performed. Reagent with antibodies (4 µL) was added to 200 µL of the supernatant adjusted to 1 × 10^6^ cells/mL. Then, samples were measured on a flow cytometer.

### Statistical analysis

Data are expressed as mean values ± SD. Survival of tumor-bearing animals during GM1-Ptx treatments was analyzed by a Kaplan-Meier survival test. The differences in qualitative variables between treatment groups on end day against control were determined by the unpaired form of the Student’s t-test. In all cases, P < 0.05 was considered to be significant .
